# Impact of Inhibition of the Mitochondrial Pyruvate Carrier on the Tumor Extracellular pH as Measured by CEST-MRI

**DOI:** 10.3390/cancers13174278

**Published:** 2021-08-25

**Authors:** Chloé Buyse, Nicolas Joudiou, Cyril Corbet, Olivier Feron, Lionel Mignion, Julien Flament, Bernard Gallez

**Affiliations:** 1 Louvain Drug Research Institute, Biomedical Magnetic Resonance, Université catholique de Louvain (UCLouvain), 1200 Brussels, Belgium; chloe.buyse@uclouvain.be (C.B.); lionel.mignion@uclouvain.be (L.M.); 2 Louvain Drug Research Institute, Nuclear and Electron Spin Technologies (NEST) Platform, Université catholique de Louvain, 1200 Brussels, Belgium; nicolas.joudiou@uclouvain.be; 3 Pole of Pharmacology and Therapeutics (FATH), Institut de Recherche Expérimentale et Clinique (IREC), Université catholique de Louvain (UCLouvain), 1200 Brussels, Belgium; cyril.corbet@uclouvain.be (C.C.); olivier.feron@uclouvain.be (O.F.); 4 Commissariat à l’Energie Atomique et aux Energies Alternatives (CEA), Centre National de la Recherche Scientifique (CNRS), Molecular Imaging Research Center (MIRCen), Laboratoire des Maladies Neurodégénératives, Université Paris-Saclay, 92265 Fontenay-aux-Roses, France; julien.flament@cea.fr

**Keywords:** pH, acidosis, glycolysis, tumor, mitochondrial pyruvate carrier (MPC), MRI, CEST, ^31^P-NMR, imaging, biomarker

## Abstract

**Simple Summary:**

Dysregulated metabolism is a key hallmark of cancer cells and many solid tumors are acidic. Acidosis is responsible for cancer aggressiveness and for resistance to several treatments. In the present study, we evaluated to which extent tumor acidosis was influenced upon inhibition of the import of pyruvate into the mitochondria, the powerhouse of the cell. Using advanced molecular imaging to measure non-invasively the acidity in a model of breast cancer, we found that while some tumor regions became much more acidic, others did not show any change. This study highlights the capacity of this advanced technology to reveal the heterogeneity of response to the treatment.

**Abstract:**

(1) Background: The acidosis of the tumor micro-environment may have profound impact on cancer progression and on the efficacy of treatments. In the present study, we evaluated the impact of a treatment with UK-5099, a mitochondrial pyruvate carrier (MPC) inhibitor on tumor extracellular pH (pHe); (2) Methods: glucose consumption, lactate secretion and extracellular acidification rate (ECAR) were measured in vitro after exposure of cervix cancer SiHa cells and breast cancer 4T1 cells to UK-5099 (10 µM). Mice bearing the 4T1 tumor model were treated daily during four days with UK-5099 (3 mg/kg). The pHe was evaluated in vivo using either chemical exchange saturation transfer (CEST)-MRI with iopamidol as pHe reporter probe or ^31^P-NMR spectroscopy with 3-aminopropylphosphonate (3-APP). MR protocols were applied before and after 4 days of treatment; (3) Results: glucose consumption, lactate release and ECAR were increased in both cell lines after UK-5099 exposure. CEST-MRI showed a significant decrease in tumor pHe of 0.22 units in UK-5099-treated mice while there was no change over time for mice treated with the vehicle. Parametric images showed a large heterogeneity in response with 16% of voxels shifting to pHe values under 7.0. In contrast, ^31^P-NMR spectroscopy was unable to detect any significant variation in pHe; (4) Conclusions: MPC inhibition led to a moderate acidification of the extracellular medium in vivo. CEST-MRI provided high resolution parametric images (0.44 µL/voxel) of pHe highlighting the heterogeneity of response within the tumor when exposed to UK-5099.

## 1. Introduction

Dysregulated metabolism is a key hallmark of cancer cells. Glucose fermentation through glycolysis even in the presence of oxygen (aerobic glycolysis) is a common feature of malignant tumors [1,2,3,4]. This exacerbated glycolytic flux in tumors is leading to extracellular acidosis that is known to stimulate tumor migration and invasiveness [1,2,3,4]. In addition, acidosis in the tumor microenvironment is associated with the modulation of activity of anti-cancer agents [4,5,6]. Clinical studies have shown that an acidic microenvironment results in less favorable prognosis associated with metastatic potential and drug resistance [7].

**Figure 1:**
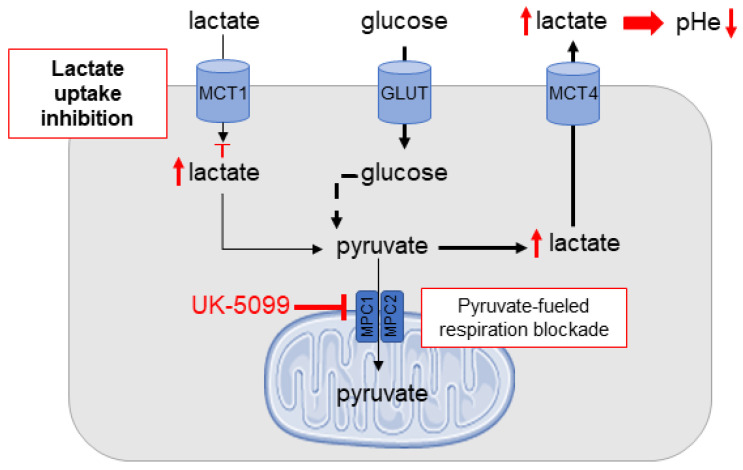
Model depicting the mechanism of extracellular acidification upon MPC inhibition.

Among the modulators of tumor metabolism, mitochondrial pyruvate carrier (MPC) has recently received particular attention. MPC is located in the mitochondrial inner membrane and is responsible for importing pyruvate, the end-product of glycolysis, from the cytosol to the mitochondrial matrix. MPC is Janus-faced regarding cancer progression [8,9,10]. Some cancers exhibit partial to complete loss of MPC expression that is associated with increased cell proliferation and stem cell marker expression [9,11]. It has actually been shown that MPC disruption could promote cancer progression in colon cancer [11,12], prostate cancer [13,14], and kidney cancer [15,16]. Conversely, cancers that rely on mitochondrial pyruvate utilization to maintain growth are negatively impacted by MPC disruption [9]. For example, in breast cancer, studies showed that chemical or estrogen-related receptor alpha (ERRα)-mediated disruption of the MPC inhibited proliferation in cell lines [9,17,18]. The chemical inhibition of the MPC also decreased the growth of the SiHa cervix cancer cell line [19]. It was also found that the inhibition of mitochondrial respiration induced by bona fide MPC inhibitors 7ACC2 and UK-5099 led to the alleviation of tumor hypoxia and the radiosensitization of the FaDu tumor model [19]. In the same study, it was also found that the extracellular acidification rate (ECAR) measured in vitro was increased after exposure of SiHa cells to 7ACC2. As pyruvate cannot undergo oxidative phosphorylation (OXPHOS) in the mitochondria, there is an accumulation of pyruvate that is transformed into lactate, inhibiting the import of lactate from the extracellular medium and favoring the export of lactate through MCT4 (Figure 1).

**Figure 2:**
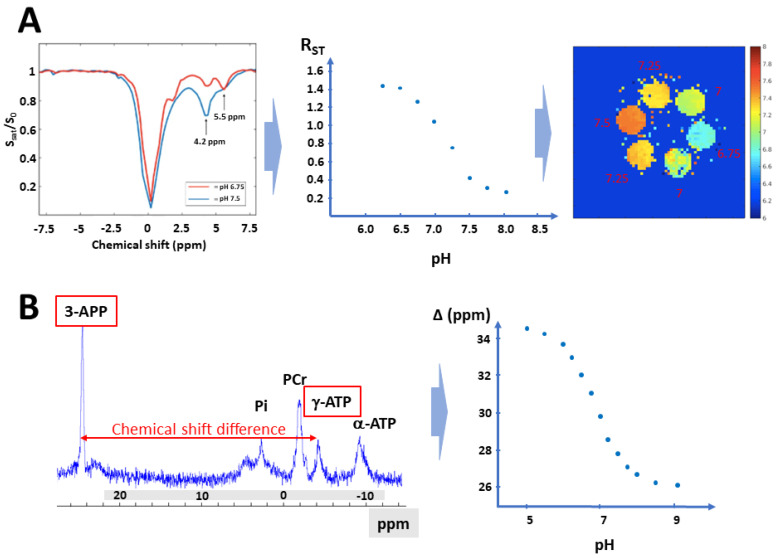
Principle of two popular MR methods to assess pHe. (**A**): CEST-MRI is based on the transfer of saturation from the exchangeable proton to the water proton reducing the MRI signal amplitude of water. Left: Z-spectrum: normalized water intensity (S/S_0_) vs. off-resonance frequency of the saturating RF for iopamidol in buffered solutions at pH 6.75 and 7.5. Center: calibration of the saturation transfer as a function of pH. Right: the calibration of the saturation transfer as a function of pH provides parametric map of pH as illustrated in tubes equilibrated at different pH. (**B**): Left: typical ^31^P-NMR spectrum recorded in a tumor. The pHe is estimated by measuring the difference in chemical shift between the signal coming from 3-APP used as extracellular pH reporter probe and the signal coming from on phosphorous present on ATP. Right: calibration of the difference in chemical shift between 3-APP and γ-ATP as a function of pH.

Because acidosis within the tumor micro-environment may have profound impact on cancer progression and treatment efficacy, this last observation prompted us to further explore the effect of MPC inhibition on the extracellular pH (pHe) in vivo. To reach this goal, we have used two non-invasive nuclear magnetic resonance methods [20]. Among magnetic resonance imaging (MRI) modalities, the chemical exchange saturation transfer (CEST) approach has emerged as a technology that provides high spatial resolution and sensitivity for in vivo imaging of tumor acidosis [20,21,22]. For this purpose, hydrophilic X-ray iodinated contrast media that are confined in the extracellular medium can be used for CEST-MRI [20,21,22]. Due to the natural process of chemical exchange of labile protons between molecules, these protons on the CEST agent are transferred to a nearby water molecule. This transfer of saturation from the exchangeable proton to the water proton reduces the MRI signal amplitude of water. Iopamidol possesses two amide proton pools that can be saturated at 4.2 and 5.5 ppm (Figure 2A). A ratiometric procedure allows to measure pHe independently on the concentration of iopamidol in the pH range of 6.0 to 8.0. This technique has been used in a variety of tumor models [23,24,25,26,27] and in cancer patients [28,29]. The technique has also been used to monitor the changes in pHe in tumors induced by treatments such as administration of bicarbonate [30,31] or after the pyruvate dehydrogenase kinase inhibitor dichloroacetate [32]. In parallel, we also exploited ^31^P-NMR spectroscopy using 3-aminopropylphoshonate (3-APP) that has long been used to assess pHe in different tissues and especially in tumors [33,34,35,36] (Figure 2B).

In the present study, we evaluated the effect of the MPC inhibitor UK-5099 on extracellular acidification. First, we observed in vitro that the exposure of SiHa (cervix cancer) and 4T1 (breast cancer) cell lines to UK-5099 led to an increase in extracellular acidification rate. Using CEST-MRI, we found that daily treatment with UK-5099 led to a significant decrease of 0.22 pHe units in mouse 4T1 tumors. The response was rather heterogenous within tumors with a shift towards acidification for about 16% of the voxels. This change in pHe was not detectable using ^31^P-NMR spectroscopy using 3-APP as pHe reporter.

## 2. Materials and Methods

### 2.1. Cell Culture

The 4T1 breast cancer cells were cultured at 37 °C in a humidified atmosphere with 5% CO_2_ and maintained in RPMI-1640 medium (Thermo Fisher Scientific, Merelbeke, Belgium) supplemented with 10% heat-inactivated FBS (Thermo Fisher Scientific). SiHa cervix cancer cells were maintained in DMEM supplemented with 10% heat-inactivated FBS. Both cell lines were acquired from ATCC where they are regularly authenticated by short tandem repeat profiling. All cell lines were tested for mycoplasma contamination with the PCR-based MycoplasmaCheck assay (Eurofins, Ebersberg, Germany) before being used.

### 2.2. Lactate Production and Extracellular Acidification Rate (ECAR)

For glucose and lactate dosage, cells (2 × 10^5^ cells/well; 3 wells/condition) were seeded in 12-well plates with 2 mL of their routine culture medium. After 24 h, medium was replaced by 500 µL of DMEM pH 7.4 containing 10 mmol/L D-glucose and supplemented with 2 mmol/L L-glutamine and 10% dialyzed FBS (Sigma-Aldrich, Overijse, Belgium), in presence of 10 µmol/L UK-5099 (Sigma-Aldrich) or vehicle (DMSO; Sigma-Aldrich). Initial concentrations of glucose and lactate in the experimental medium were also assessed by including control wells containing only cell culture medium (no cells) on each plate. After incubation for 24 h, extracellular media were collected and deproteinized by centrifugation (15 min, 10,000 rpm, 4 °C) in 10 kDa cut-off filter tubes (VWR). Glucose and lactate concentrations were measured in the samples (50 µL) by using enzymatic assays (CMA Microdialysis AB, Kista, Sweden) and a CMA 600 analyzer (Aurora Borealis, Solna, Sweden). Data analysis was done by calculating the difference in metabolite concentrations between the control wells and the experimental wells. Data were then normalized by the protein content in each well and expressed in µmol/h/mg protein.

Oxygen consumption rate (OCR) and extracellular acidification rate (ECAR) were measured by using the Seahorse XF96 analyzer (Agilent, Santa Clara, CA, USA). Briefly, cells (1.5 × 10^4^ cells/well; 6 wells/condition) were seeded in Seahorse 96-well cell culture plates in their routine culture medium, in presence of 10 µmol/L UK-5099 or vehicle (DMSO, Sigma-Aldrich). After 24 h, medium was replaced by 175 µL unbuffered serum-free DMEM pH 7.4 supplemented with 2 mmol/L L-glutamine, still in presence of the treatment. OCR and ECAR values were assessed before (3 cycles of 3 min mixing/4 min measuring) and after (4 cycles of 3 min mixing/4 min measuring) the injection of 10 mmol/L D-glucose. Glucose-dependent ECAR was calculated by comparing the values before and after addition of the substrate. Data were normalized by the protein content in each well and expressed in pmol/min/µg protein or mpH/min/µg protein for OCR and ECAR, respectively.

### 2.3. Tumor Model

All experiments involving animals were performed in accordance with the Belgian law concerning the protection and welfare of the animals and were approved by the UCLouvain ethics committee (Agreement reference: 2018/UCL/MD/021).

**Figure 3:**
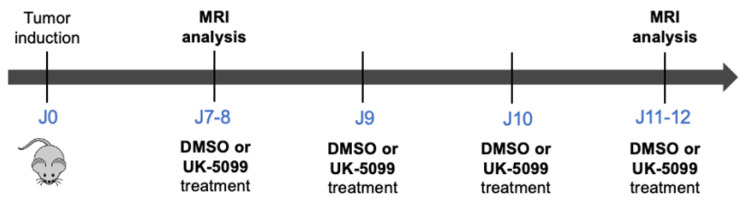
Experimental protocol to assess the effect of UK-5099 on extracellular pH in vivo.

The 2 × 10^5^ 4T1 cells in 100 µL of PBS were injected intramuscularly in the right hind paw of 15-week-old female BALB/c JNRj mice. On Day 7 or 8, when tumors reached a volume of about 200 mm^3^ (real values measured by MRI were 203 ± 14 mm^3^), the pHe of tumors was measured and found comparable in both groups (control or treated). Mice were treated with daily intraperitoneal injection of UK-5099 (3 mg/kg) or vehicle for 4 days. Tumors were last analyzed on Day 10 or 11 after induction (Figure 3).

### 2.4. CEST-MRI Experiments

- CEST calibration in vitro

All experiments were performed on a 11.7T Biospec MRI (Bruker, Ettlingen, Germany). Solutions containing 30 mmol/L of Iopamidol (Iopamiron 300, Bracco, Milan, Italy) and HEPES were set at 9 different pH (from 6.25 to 8). Calibrations were done at 37 °C. CEST acquisitions were acquired with a 40-mm ^1^H volume coil. The CEST sequence was a RARE sequence (TR = 4.0 s, TE = 23.0 milliseconds, rare factor = 10, slice thickness = 2 mm, FOV = 30 mm, matrix size = 64 × 64, in-plane special resolution = 468 µm) with an additional continuous 4 µT irradiation for 4 s. The Z spectrum was acquired between −8 to 8 ppm (81 frequencies) for a total acquisition time of 27 min 20 s.

- In vivo analysis

Mice were anesthetized with Isoflurane (Isoflo, Zoetis, Zaventem, Belgium) and placed in a device allowing their analysis and maintenance of the anesthesia at 1–2% Isoflurane. Their breathing was monitored by a pressure sensor placed in the abdomen for the entire duration of the experiment and their temperature was monitored and maintained at 37 °C by a blanket heater connected to a water bath. Iopamidol was injected intravenously through the tail vein (3 g Iodine/kg). Mice were placed in a 40-mm ^1^H volume coil. CEST images were acquired using the same parameters as in vitro experiment. Acquisition were started 20 min after the injection. All voxels were analyzed using a custom-written script in Matlab (The MathWorks Inc., Natick, MA, USA). The Z-spectra were centered on the bulk water signal to correspond to the zero frequency. Values were measured at the frequency offsets of 4.2 and 5.5 ppm and used in the Equation (1):

ST = (S_−Δω_ − S_+Δω_)/S_0_ (1)

where S = signal intensity at 4.2 or 5.5 ppm and S_0_ = bulk water signal intensity without saturation. Saturation transfer values at 4.2 and 5.5 ppm can be used to calculate their ratio (2):

RST = (ST 5.5)/(ST 4.2)(2)

In order to eliminate outliers, different filters were applied to the data. A threshold of 2% was used to discriminate between enhanced and not enhanced voxels. Negative ST values were excluded as well as RST values above 0.2 and below 1.55. pH values were calculated based on the RST and the corresponding pH values of the calibration curve. pH maps were obtained by overlaying anatomical images and pH values in each voxel. Only tumors giving a pH map including more than 10 voxels (volume = 0.44 µL) were kept for the statistical analysis.

### 2.5. P-NMR Spectroscopy

- pH Calibration in vitro

Solutions were prepared with 10 mM 3-APP, 10 mM ATP and HEPES (Sigma-Aldrich) and adapted to 15 different pH values (from 5 to 9). pH values were measured using a pH-meter (InoLab pH 730 WTW, Weilheim, Germany). Solutions were placed in the setting later used for the in vivo studies. The values used for the calibration were studied at 37 °C. A RARE sequence was followed by a ^31^P single pulse sequence (TR = 500 ms, number of repetitions = 1024, total acquisition time = 8 min, resolution = 2.44 Hz) acquired with a ^1^H/^31^P surface coil.

- In vivo spectroscopy

Anesthetized mice were injected intraperitoneally with 300 µL of 3-APP (75 mg/mL) 30 min before analysis. Prior to spectral acquisitions, two RARE sequences were used: axial (FOV = 30 × 20 mm, matrix size = 128 × 128 mm, in-plane special resolution = 234 × 156 µm) and sagittal (FOV = 25 × 20 mm, matrix size = 128 × 128 mm, in-plane special resolution = 195 × 156 µm). Spectroscopy was done using the same sequence as for in vitro acquisition with the addition of 9 saturations slices placed according to the two anatomical RARE sequence. For in vivo acquisition, the number of repetitions was 4096 for a total acquisition time of 34 min.

- Spectra analysis

Spectrum were analyzed using TopSpin (Bruker, Ettlingen, Germany). For pH values determination, the chemical shift of γ-ATP was used as an internal reference. The pH for each spectrum was calculated according to the equation:

pH = Ao + log [(δ − δ_min_)/(δ_max_ − δ)] (3)

where Ao = 6.8672; δ_max_ = 26.0916 and δ_min_ = 34.6452

### 2.6. Statistical Analysis

Statistical analysis was performed using GraphPad Prism software (San Diego, CA, USA). pH values before and after treatments were compared by a paired *t*-Student test with *p* ≤ 0.05 considered significant. Results are represented as mean ± SE. The number of experiments is provided in each Figure.

## 3. Results

**Figure 4:**
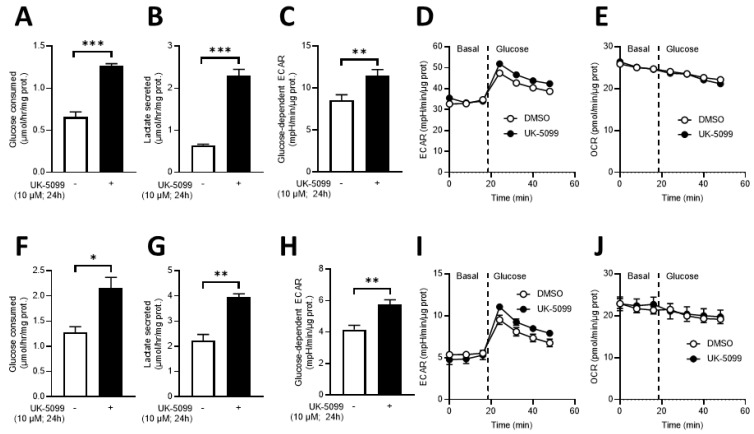
Impact of UK-5099 on metabolic parameters. SiHa (top, (**A**–**E**)) and 4T1 (bottom, (**F**–**J**)) cells were treated for 24 h with 10 μM UK-5099 in glucose-containing medium. Effect of treatment on glucose consumption (**A**,**F**), lactate release (**B**,**G**), extracellular acidification rate (ECAR) (**C**,**H**), time course of extracellular acidification (**D**,**I**) and oxygen consumption rate (OCR) (**E**,**J**). * *p* < 0.05; ** *p* < 0.01; *** *p* < 0.001.

In vitro, we observed that the exposure of cancer cells (SiHa and 4T1) to UK-5099 (10 µM) for 24 h led to a significant increase in glucose consumption and lactate release in the media as well as a significant increase ECAR as shown in Figure 4. However, there was no significant difference in OCR between control cells and cells exposed to UK-5099 (Figure 4).

**Figure 5:**
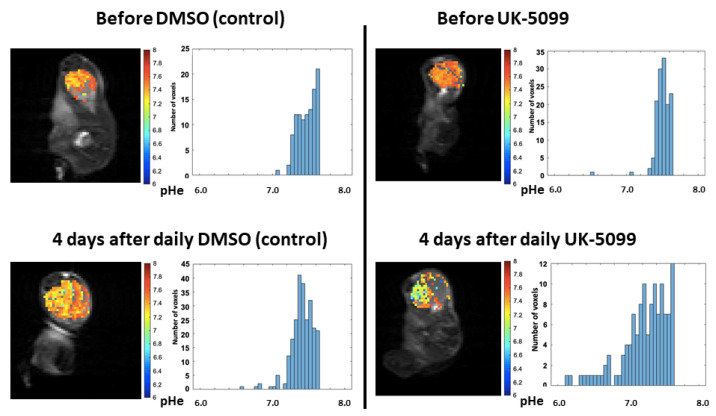
Evolution of pHe evaluated by CEST-MRI. Typical images and histograms of distribution of pHe values recorded before and after vehicle administration (**left**), before and after UK-5099 administration (**right**) (daily IP injection during 4 days, 3 mg/kg). Images are recorded on the same mice. Note that the pHe values remained rather identical in the control group while there is a clear shift in pHe towards more acidic values. Note also the large heterogeneity of pHe values recorded after the UK-5099 treatment.

For the in vivo studies, we applied an administration scheme of UK-5099 inspired from a previous work where this compound was shown to radiosensitize tumors [19]. MR protocols were applied on separate cohorts of mice for CEST-MRI and ^31^P-NMR spectroscopy to avoid any possible interference between the pH reporter probes administered before the MR analysis. The MR protocols were applied before and after 4 days of treatment. Representative distribution of pHe within tumors before and after 4 days of treatment are presented in Figure 5.

**Figure 6:**
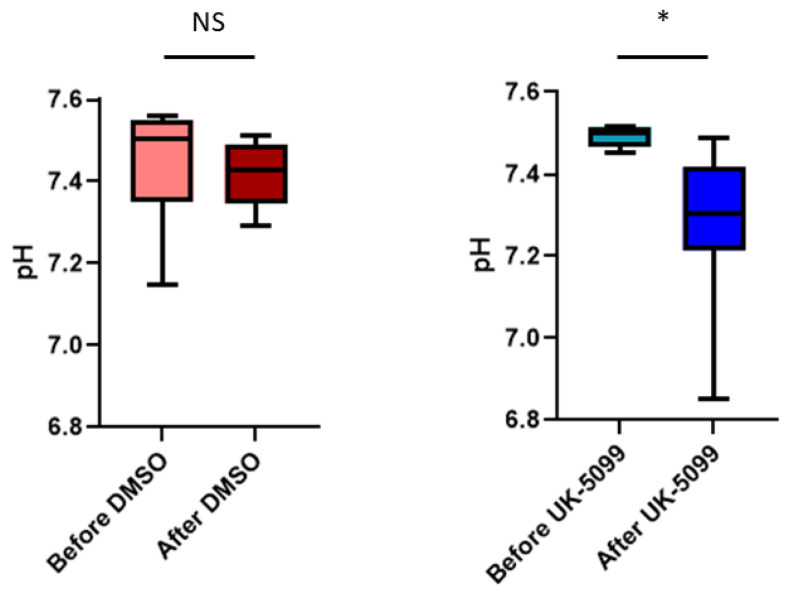
Evolution of pHe (estimated by CEST-MRI) in 4T1 tumors in mice before and after treatment 4 days with DMSO (control) or UK-5099. NS: not significant; *: *p* < 0.05 (paired *t*-test, *n* = 8 in the DMSO group and *n* = 8 in the UK-5099 group).

The analysis of the pooled data recorded on 8 tumors in each group shows that there was no significant change (*p* > 0.05), neither in the mean or in in the median pHe values in the mice injected daily with the vehicle. In contrast, pHe was significantly decreased (*p* < 0.05) in mice daily treated with UK-5099 (Figure 6), with a difference of 0.22 pHe units for the mean values, and 0.20 pHe units for the median values.

**Figure 7:**
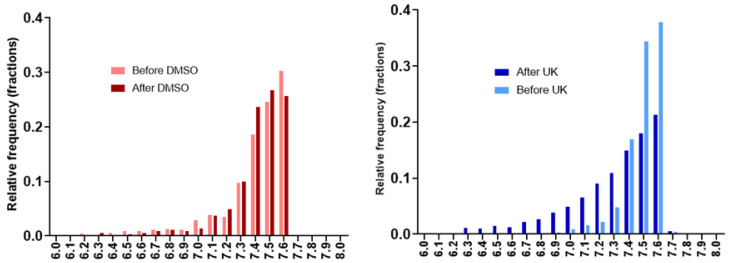
Histograms of distribution all pHe values recorded in individual voxels using CEST-MRI. **Left**: pHe values before (light red) and after (dark red) treatment during 4 days with vehicle. **Right**: pHe values before (light blue) and after (dark blue) treatment during 4 days with UK-5099.

The pooled histograms of distribution of pHe values recorded before and after treatment (*n* = 8 mice in each group) are shown in Figure 7. Values are almost superimposable in the control group while there is a clear shift of values towards acidic pHe. This is confirmed by the analysis of the skewness of the distribution which is the measure of the asymmetry of a histogram. The skewness was negative in all cases, the tail being longer on the left side (meaning lower pHe values). The tail towards more acidic values after UK-5099 is clearly seen in Figure 7 (right panel). The number of voxels with a pHe value inferior to 7.0 increased from 1.5% to 16.2% after 4 days of UK-5099 treatment. This means also that a large majority of voxels remained with a pHe superior or equal to 7.0.

**Figure 8:**
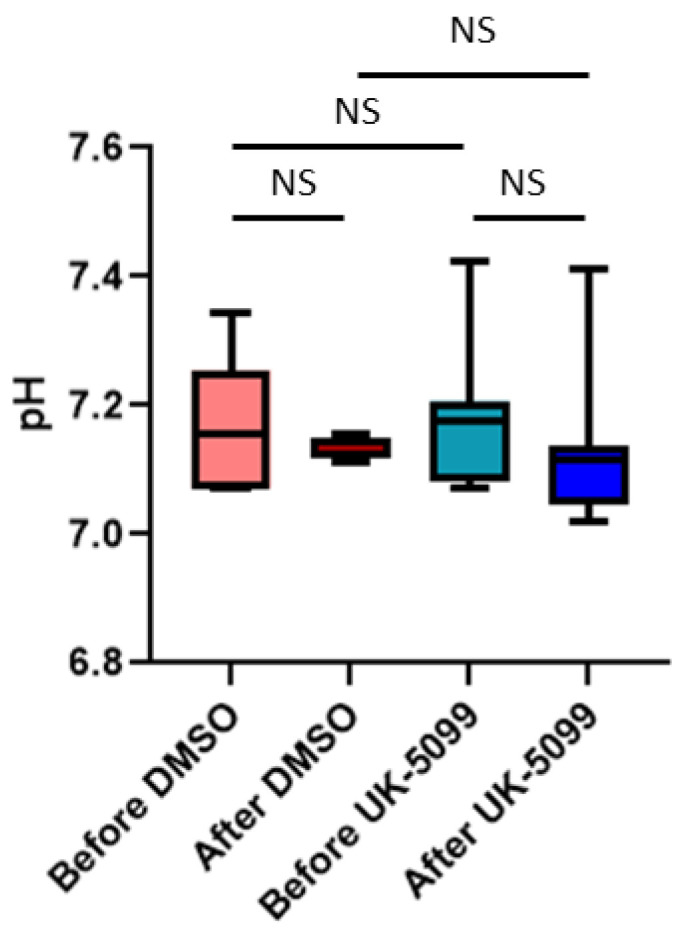
Evolution of pHe (estimated by ^31^P-NMR spectroscopy) in 4T1 tumors in mice before and after treatment 4 days with DMSO (control) or UK-5099 (NS: not significant, paired *t*-test, *n* = 8 for each group).

We also tested the evolution of pHe using ^31^P-NMR spectroscopy using 3-APP as pHe reporter. With this method, we did not find any significant change in pHe values in control and UK-5099 treated mice (Figure 8).

## 4. Discussion

The objective of this study was to evaluate the impact of the MPC inhibitor UK-5099 on the pHe in tumors. Previous observations have shown that 7ACC2, another MPC inhibitor, increased ECAR in several cancer cell lines and that UK-5099 increased lactate release by cancer cells in the presence of glucose [19]. It was also found that the inhibition of cellular lactate influx was an indirect consequence of intracellular pyruvate accumulation [19]. We have first evaluated in vitro the glucose consumption, the lactate secretion and the ECAR in the cervix cancer cell line SiHa and the triple negative breast cancer cell line 4T1. The extents of glucose consumption and lactate release were higher at the basal level for 4T1 cells than for SiHa cells. We observed that the glucose consumption, lactate secretion and acidification rate were significantly increased upon exposure of the cells to UK-5099 10 µM (Figure 4). We did not observe any change in OCR due to UK-5099 exposure (Figure 4). The presence of glutamine in the medium may account for this observation as the absence of change in oxygen consumption may be due to an increase in glutamine utilization. We next aimed to explore the effect of UK-5099 on mouse 4T1 breast tumors in vivo. CEST-MRI revealed that the pHe was significantly decreased after the administration of UK-5099 during 4 days (Figure 6). This effect was rather modest with a shift of 0.22 pHe unit towards acidification. ^31^P-NMR spectroscopy using 3-APP that is measuring pHe over the whole tumor did not show any significant effect of the treatment (Figure 8). Of note, the presence of residual muscle tissue in the volume sampled by ^31^P-NMR spectroscopy (as observed by the presence of phosphocreatine in the spectrum, Figure 2) may also account for the inability to detect a pHe change when using this technique. Most importantly, the effect that we did observe using CEST-MRI was highly heterogenous within the tumors, as shown by visualizing the images (Figure 5) or by analyzing the histograms of distribution (Figure 7). Only a small proportion of voxels (16%) shifted towards acidic values (pHe < 7.0), an effect that was sufficient to shift the pHe mean and median values (Figure 6).

Considering the large in vitro effect of MPC inhibition on ECAR (Figure 4), the modest effect observed in vivo using CEST-MRI or even absence of effect using ^31^P-NMR is somehow surprising. It should be noted that a discrepancy between in vitro and in vivo responses as well as heterogeneity in responses within tumors have already been reported in several studies on tumor metabolism [37,38,39,40], highlighting the limitation of in vitro studies to truly reflect the complex tumor behavior. As far as pHe is concerned, difference in pH buffering capacity between in vitro and in vivo conditions may account for our observations. Indeed, it is likely that tumors exhibit a higher buffering capacity since circulating blood can remove the excess H^+^ whereas H^+^ released from cultured cancer cells accumulate in the dish. Temporal and regional fluctuations in tumor perfusion may actually also account for the observed heterogeneity in “acidic voxels” upon mouse treatments with UK-5099.

It should also be emphasized that cancer cells do not represent the majority of cells present in the tumor microenvironment (TME). In addition to non-cellular components of extracellular matrix, the TME consists of tumor cells, stromal fibroblasts, endothelial cells and immune cells like microglia, macrophages and lymphocytes [41,42]. Presently, the effect of MPC blockade has not been investigated on all types of cells composing the TME, and we may expect a different impact of treatment on these non-cancerous cells. Another possible contributor to this lower acidification of the TME could be a compensatory mechanism occurring when tumors are treated chronically with an MPC inhibitor. Tumor cells indeed exhibit extraordinary metabolic plasticity to adapt to constraints or changes in their environment [6,43,44,45,46]. It has been previously demonstrated that cells may tolerate loss of MPC activity by metabolic rewiring to compensate for the loss of pyruvate oxidation [8]. Among compensatory mechanisms, increase in glutamine utilization [47,48] and pyruvate-alanine cycling [49] have been described when mitochondrial pyruvate transport is impaired. If present, those mechanisms may lead to a lower glycolysis utilization or less transformation of pyruvate into lactate with a consequent lower effect on extracellular acidification.

Finally, our study highlights the high value of using a high-resolution imaging (0.44 µL/voxel) of pHe for monitoring the effect of anti-cancer drugs. While ^31^P-NMR spectroscopy did not detect any significant change in pHe after UK-5099 treatment (Figure 8), CEST-MRI did reveal a large heterogeneity in response to the exposure to the MPC inhibitor. More importantly, image analysis and histograms distributions of pHe values showed that a small part of the tumors were affected by extracellular acidification (Figure 5 and Figure 7). As discussed earlier, this heterogeneity of response may be the consequence of differences in regional perfusion, distinct responses coming from different cells present in the TME but also from compensatory mechanisms. Further investigation is warranted to identify the most relevant factors influencing pHe.

Of note, the pHe values evaluated before treatment were slightly lower when estimated by ^31^P-NMR than with CEST-MRI. While ^31^P-NMR measures the pHe over the whole tumor, our CEST-MRI protocol was done on a single slice through the center of the tumor. Differences in position may possibly account the difference observed between both techniques. It is also worth to mention that the basal pHe values (before treatment) measured in our study for the 4T1 tumor model were higher than 7.0 (for CEST-MRI and ^31^P-NMR) while a recent study that compared several breast cancer cell lines suggested lower pHe values for the same model (6.96 ± 0.03) [27]. Different experimental conditions may potentially explain these differences: different conditions of cell culture before inoculation and time-window for the MRI study during the tumor growth. We also noticed that the anesthesia used was different: ketamine/xylazine in the reference study [27] and isoflurane in the present study. As ketamine/xylazine is known to induce a rapid drop in tumor perfusion and oxygenation [50,51,52], we hypothesize that this experimental factor may potentially contribute to subtle difference in glycolytic activity and pHe values recorded for the same tumor model. A possible factor that may also account for differences between the pHe measured and data from the literature relies on our CEST measurements that were done only on post-contrast images as described in [23,30]. More recent papers have used the difference between the CEST contrast after iopamidol injection and the CEST contrast before iopamidol injection, allowing the measurement of the sole CEST contribution due to iopamidol and removing the endogenous one [27,28,31]. We cannot exclude that that the protocol based on the sole post-iopamidol CEST imaging has led to an overestimation of pHe values. In the future, it would be interesting to further evaluate the effect of MPC inhibitors with other pHe imaging modalities such as biosensor imaging of redundant deviation in shifts (BIRDS). BIRDS is a rapid chemical shift imaging technique in which paramagnetically shifted non-exchangeable protons on DOTA-based macrocyclic complexes are directly detected. While BIRDS is limited so far to pre-clinical studies because the sensors are not yet approved by health agencies, it could be interesting to analyze the impact of MPC inhibitors as the technique has demonstrated its value in cancer animal models [53,54,55,56].

## 5. Conclusions

MPC inhibition led to a moderate acidification of the extracellular medium in vivo. CEST-MRI provided high resolution parametric images of pHe highlighting the heterogeneity of response within the tumor when exposed to UK-5099. Our study adds to the previous demonstration on the utility of longitudinal monitoring of tumor acidosis during anti-cancer treatments [30,31,32]. As the feasibility of using CEST-MRI has already been demonstrated in patients for pH measurement [28,29,57,58], it is likely that this technology could be useful as imaging biomarker for guidance of treatments targeting tumor metabolism.

## Data Availability

Rough data available on request.
